# Prolonged Intractable Hiccups Associated with HSV (I&II) Esophagitis and *H. pylori* Gastritis

**DOI:** 10.1155/2023/3561895

**Published:** 2023-02-28

**Authors:** Christina Hopkins, Asha Bansari, Michael Ladna, Bahram Dideban

**Affiliations:** Department of Internal Medicine, University of Florida, Gainesville, FL, USA

## Abstract

An immunocompetent male presented with an intractable hiccup. EGD revealed circumferential ulceration of middistal esophagus and biopsies confirmed HSV (I&II) esophagitis and *H. pylori* gastritis. He was prescribed triple therapy for *H. pylori* and acyclovir for HSV esophagitis. HSV esophagitis and *H. pylori* should be included in differential for intractable hiccups.

## 1. Background

Hiccups are a common occurrence that everyone has experienced at least once in their life. Hiccups are typically a brief nuisance that self-resolve. Hiccups become particularly bothersome when they are persistent, lasting more than 48 hours. This is a rare occurrence but can be an indication of an underlying pathology. Some conditions that can cause intractable hiccups include infections in the central nervous system (CNS), pharynx, larynx and lung, arteriovenous malformations, stroke (both ischemic and hemorrhagic), malignancy, myocardial infarction, pericarditis, gastric or esophageal distention, gallbladder pathology, peptic ulcer disease (PUD), esophagitis, gastritis, small bowel obstruction (SBO), alcohol use, and electrolyte disturbances [1]. Identifying and treating the underlying cause of intractable hiccups is the best way to resolve the hiccups themselves.

HSV esophagitis is most commonly due to HSV type 1 and is generally seen in the immunocompromised [2]. Rarely, HSV type 2 occurs in an immunocompetent host [2]. The most common symptoms include acute odynophagia, dyspepsia, and fever [3], while epigastric pain, dysphagia, and prodrome of flu-like symptoms are less common [4]. Although HSV-2 esophagitis in an immunocompetent host has been reported in two previous cases [2, 4], the presenting complaints in those cases were more typical and included odynophagia and dyspepsia. Per review of available literature, there have been no reported cases of HSV (I and II) esophagitis and *H. pylori* infection in an immunocompetent adult with a presenting complaint of intractable hiccups.

## 2. Case Presentation

An obese male in his 50s with a past medical history of hypertension (HTN), gastroesophageal reflux (GERD), and chronic kidney disease (CKD) stage 3a presented to the emergency department (ED) with intractable hiccups. He did not try anything to alleviate the hiccups nor could he identify any aggravating factors. He presented to the ED a total of 4 times since the onset of the hiccups which first started 1 week before the first ED visit. On the first ED visit, he reported a chief complaint of hiccups associated with nausea and vomiting. Admission to the hospital was offered, but the patient declined and was discharged from the ED. He returned 2 days later with hiccups associated with abdominal pain and was discharged from the ED after abdominal cross-sectional imaging was unremarkable. He returned 1 day later for hiccups but was discharged from the ED after cross-sectional imaging of the brain showed no intracranial abnormalities. He presented to ED a final time 1 day later. On the fourth ED visit, the initial examination showed sinus tachycardia to 107 bpm. The remainder of the physical exam was unremarkable. The initial lab work was remarkable for hyponatremia with serum sodium of 122 mEq/L and an acute kidney injury (AKI) with a serum creatinine of 2.24 mg/dL up from a baseline of 1.7 mg/dL. His incessant hiccupping would lead to vomiting, so he had very little oral intake throughout the duration of this period and thus was unable to take his acid reflux medication. He did not have any personal history of cancer, liver disease, or peptic ulcer disease (PUD). He was an active smoker with a 4-year pack history but denied any alcohol or recreational drug use. He was heterosexual and sexually active with a single female partner; however, it was not clarified whether he had engaged in orogenital sexual activity.

**Figure 1:**
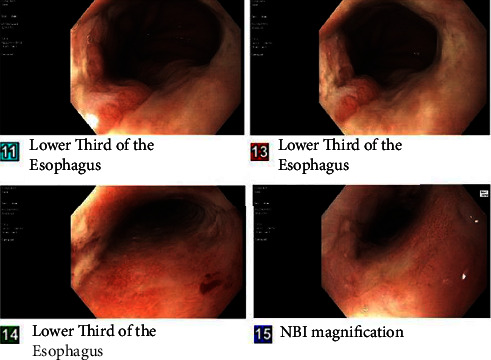
Upper endoscopy showing superficial ulcer at the distal esophagus.

Since the patient had presented multiple times to the emergency department with the same complaint, several imaging studies were already obtained. CT angiogram of the head and neck was negative for any abnormality. CT of the abdomen and pelvis with and without contrast showed a moderate hiatal hernia, but no evidence of pancreatitis, gastric distention, or small bowel obstruction. CT chest revealed abnormal thickening of the distal one-third of the esophagus; thus, gastroenterology was consulted. An esophagogastroduodenoscopy (EGD) showed a circumferential superficial ulcer at the distal esophagus, accompanied by inflammation, and decreased vascularity that spanned to the midesophagus, a 2 cm hiatal hernia, and erythematous mucosa in the stomach and duodenal bulb (Figure 1). Biopsies were obtained from the esophagus and stomach. Biopsies of the stomach were positive for *H. pylori*, and immunostaining of the esophageal tissue was positive for herpes simplex virus (HSV I & II). The pathology resulted after the patient was discharged, so further serology was unable to be obtained. He tested negative for HIV.

The patient's hiccups improved initially in the emergency department with baclofen, thorazine, gabapentin, famotidine, and oral lidocaine, but they did return several times throughout his admission. He was discharged on a combination of pantoprazole, baclofen, and gabapentin, which seemed to provide the most relief. The patient was contacted after discharge and informed of the biopsy results. He was prescribed a 14-day course of bismuth subcitrate, metronidazole, and tetracycline for H. pylori gastritis and a 10-day course of acyclovir for HSV esophagitis.

A posthospital telephone call was made a few days after discharge, and the patient reported his hiccups had completely resolved. The patient followed up with his PCP about 6 weeks after discharge. At that appointment, he discussed the difficulty in affording the combination pill of bismuth subcitrate, metronidazole, and tetracycline due to lack of insurance and as such had not yet started any treatment for H. pylori. At that time, he was switched to triple therapy which consisted of pantoprazole, amoxicillin, and clarithromycin twice daily. The patient was scheduled for a repeat EGD 8 weeks after discharge but was unfortunately lost to follow-up.

## 3. Discussion

This case demonstrates the rare finding of multiple infections of the GI tract with the alerting symptom of intractable hiccups. The occurrence of peptic ulcer disease (PUD) and gastritis secondary to H. pylori is well known, as is its somewhat common presenting symptom of hiccups. But hiccups related to HSV esophagitis are much less common. Only 3 cases of HSV esophagitis were found where the presenting complaint was hiccups [5–7], and only one occurred in an immunocompetent patient [5].

There have been many cases of isolated H. pylori infections or HSV esophagitis, but the coexistence of both H. pylori and HSV is much rarer. Few cases were identified, one in which an immunocompetent adolescent had positive serology IgM and IgG for both HSV 1 and 2, with active esophageal ulcers and gastric inflammation consistent with H. pylori gastritis [8]. This case, however, presented with fever, odynophagia, and chest pain. Another case found HSV-1 and H. pylori within the same prepyloric ulcer [9].

The explanation behind the coexistence of these infections is currently only theorized. A study conducted by Löhr, Nelson, and Oldstone [9] found the presence of HSV-1 within the bordering cells of peptic ulcers and postulated how HSV-1 infection within the stomach occurred [9]. It is thought that HSV initially enters the vagal ganglia through the oropharynx or peripheral sites and remains dormant until an activating event occurs [9]. An activating event can be any immunosuppression or inflammation within a site that receives stimulation via the vagus nerve. Once activated, HSV can then travel along the vagus nerve and spread to the target site, causing more inflammation and ulceration [9]. The alternative explanation is that of primary infection. In our case, reactivation seems most likely, although primary infection cannot be ruled out. It is possible that the H. pylori infection was the activating event for the HSV infection. The issue with this explanation is that it would be more likely that both infections would be collocated in the same area. Alternatively, this patient had a known history of acid reflux which was likely poorly controlled due to hiatal hernia, current tobacco use, and poor intake of medications from the intractable hiccups. This reflux could have produced enough inflammation to induce the reactivation of HSV and the subsequent formation of esophageal ulceration. The *H. pylori* infection in this case may have been an incidental finding.

The rarity of coinfection may be due to a proposed protective association between *H. pylori* against HSV. According to Tsamakidis et al. [10], there is an inverse relationship between the occurrence of *H. pylori* infection and HSV-1 in gastric and duodenal ulcers among 90 participants [10]. Krasteva and Petrova found a similar association, in which six participants with positive serology for acute HSV-2 infection and seven of eight participants with positive HSV-1 IgM were negative for *H. pylor*i [11].

It is interesting that at the time of the posthospital follow-up call, the patient's hiccups had completely resolved before the treatment for *H. pylori* was initiated. What then explains the cause of his hiccups? It is possible that they were due to uncontrolled acid reflux or the presence of the esophageal ulcer itself. The initiation of PPI therapy while inpatient promoted healing of the ulcer and suppression of GERD. However, it is unlikely that significant ulcer healing could have occurred within only a week or so of PPI use. Perhaps the HSV infection is to blame. In the immunocompetent, it is often a self-limited infection and generally resolves within several weeks [12], but the addition of acyclovir can hasten the process. Thus, the etiology was likely multifactorial, a combination of esophageal irritation due to esophageal ulcer and uncontrolled acid reflux, HSV infection, and gastritis secondary to *H. pylori*.

Although both HSV esophagitis and *H. pylori* gastritis should be appropriately treated with acyclovir and triple therapy, respectively, the initiation and completion of these therapies do not appear to be necessary to induce short-term relief of hiccups which can be done via direct symptomatic control with medications such as baclofen, gabapentin, and PPI.

A common finding in severe reflux esophagitis is inflammation, ulceration, and decreased vascularity within the distal esophagus. These endoscopic findings could also indicate malignancy or infection and thus should be biopsied. Any presence of middistal esophageal inflammation or ulceration should also prompt the clinician to obtain IgM and IgG serology for HSV and an HIV screen, preferably prior to discharge if the procedure is performed inpatient. This case demonstrated the importance of pursuing upper endoscopy when presented with intractable hiccups and should broaden a clinician's differential to include HSV esophagitis as an underlying etiology.

## Data Availability

The data used to support the findings of this study are available from the corresponding author upon request.

## Abbreviations

CKD: Chronic kidney disease
CNS: Central nervous system
CT: Computed tomography
EGD: Endoscopy
GERD: Gastrointestinal reflux disease
*H. pylori*: *Helicobacter pylori*
HIV: Human immunodeficiency virus
HSV: Herpes simplex virus
HTN: Hypertension
PCP: Primary care physician
PPI: Proton pump inhibitor
PUD: Peptic ulcer disease
SBO: Small bowel obstruction.
